# The relationship between parental overprotection and student depression: The chain mediation role of psychological control and well-being

**DOI:** 10.1371/journal.pone.0328498

**Published:** 2025-07-18

**Authors:** Shuyu Huang

**Affiliations:** College of Teachers, Chengdu University, Chengdu, Sichuan, China; The Open University of Israel, ISRAEL

## Abstract

This study investigated the relationship between parental overprotection and adolescent depressive symptoms, focusing on the chain mediation roles of psychological control and well-being. Using a survey-based design with a convenience sample of 823 adolescents aged 10–14, data were collected through validated scales measuring parental overprotection, psychological control, well-being, and depressive symptoms. Mediation analysis, conducted with the PROCESS macro for SPSS, revealed that parental overprotection exerts a significant positive effect on adolescent depression. Specifically, parental overprotection increases psychological control, which in turn reduces well-being, thereby exacerbating depressive symptoms. These findings underscore the importance of balanced parenting practices that limit psychological control and promote adolescent autonomy and well-being. Educators and mental health professionals are encouraged to collaborate with families to reduce overprotective behaviors and enhance adolescents’ resilience against depressive symptoms.

## 1. Introduction

Adolescence is a critical period marked by profound physical, emotional, and social changes, during which individuals are increasingly vulnerable to mental health challenges, including depression. According to the World Health Organization [1], adolescent depression is a rising concern worldwide, with an estimated 13% of adolescents experiencing depressive symptoms that can interfere with their daily lives and long-term development. Adolescent depression not only disrupts personal well-being but also has wider social implications, affecting educational outcomes, peer relationships, and overall life satisfaction [2]. Recognizing the environmental factors that contribute to adolescent mental health is essential for developing effective preventive measures and therapeutic interventions.

Family dynamics, particularly parenting styles, play a pivotal role in shaping adolescents’ mental health. Parenting styles encompass various approaches and behaviors parents use to guide, support, and discipline their children [3]. Research indicates that positive parenting, characterized by warmth, responsiveness, and support, tends to foster healthy psychological development in adolescents, reducing the likelihood of depressive symptoms [4]. Conversely, negative parenting behaviors—such as excessive control, criticism, or indifference—are associated with higher levels of psychological distress, anxiety, and depressive symptoms [5]. The role of parenting styles in adolescent mental health is therefore a topic of considerable interest within the fields of psychology, education, and public health.

Parental overprotection has been identified as a particularly influential parenting style related to adolescent mental health. Defined by its emphasis on shielding children from perceived dangers and limiting their autonomy, overprotective parenting aims to prevent potential harm but may inadvertently hinder adolescents’ development of resilience, self-confidence, and autonomy [6,7]. Adolescents raised in such environments may develop a heightened dependency and reduced problem-solving skills, increasing their susceptibility to external stressors [8].

The psychological mechanisms underlying the effects of overprotective parenting on adolescent mental health have received increasing research attention in recent years [9]. Recent findings suggest that overprotection may diminish adolescents’ self-efficacy or belief in their ability to handle challenges effectively, which is critical for mental well-being [10]. Moreover, overprotective parenting may impair the development of autonomy—a fundamental aspect of adolescence—by restricting opportunities for independent decision-making and problem-solving [11]. This limitation not only affects adolescents’ self-perception but also impacts their social interactions, contributing to feelings of helplessness, low self-worth, and social anxiety [12].

Research on parental overprotection has also been conducted across diverse cultural contexts, highlighting the importance of considering cultural nuances in parenting practices [13]. In collectivistic societies, familial interdependence is often emphasized, and parental control may be perceived differently than in individualistic societies, where independence is highly valued [14]. Nevertheless, excessive parental control consistently associates with adverse adolescent psychological outcomes across cultures [15].

The growing body of research underscores the importance of understanding how different parenting styles, especially overprotection, associated adolescents’ mental health. Although much is known about the associations between parenting and psychological outcomes, less is understood about the specific pathways through which overprotective parenting may lead to depressive symptoms. Identifying these pathways can provide valuable insights for developing family-based interventions aimed at mitigating adolescent depression. Furthermore, exploring the role of psychological factors, such as self-esteem and autonomy, within the context of overprotective parenting can inform mental health professionals about critical intervention points to support adolescent well-being effectively.

In sum, with adolescent depression on the rise, understanding the role of family associated and specific parenting behaviors has never been more urgent. This study seeks to address the gaps in current literature by examining how parental overprotection may contribute to adolescent depressive symptoms, offering a comprehensive perspective that can inform future family education and mental health policies.

### 1.1. Theoretical framework

Bronfenbrenner’s ecological systems theory posits that human development unfolds through dynamic, reciprocal interactions across multiple layers of the environment—namely, the microsystem (e.g., family, school), mesosystem (interconnections among microsystems), exosystem (indirect influences such as community resources), and macrosystem (cultural norms, societal values) [16]. Recent studies emphasize the crucial role of family as a core microsystem in shaping adolescents’ emotional and behavioral development [17]. Specifically, parental behaviors can mediate how adolescents experience and respond to risks in their larger socio-cultural environment [18].

In the context of parental overprotection as an independent variable, ecological systems theory highlights how such protective behaviors are neither isolated nor static. At the microsystem level, overprotective parents may excessively monitor or restrict their children’s everyday activities, undermining adolescents’ autonomy and problem-solving abilities and thereby increasing vulnerability to stress and depressive symptoms [19]. At the mesosystem level, limited collaboration between parents and other significant environments—such as schools or peer networks—could hinder adolescents from accessing broader support systems, exacerbating the negative effects of overprotection [20]. Finally, factors in the exosystem and macrosystem (e.g., cultural norms that valorize strict parental control or social anxieties about safety) can reinforce overprotective tendencies, ultimately intensifying the risk of adolescent maladjustment [21]. Thus, ecological systems theory underscores the notion that parental overprotection operates within a multifaceted social and cultural context, influencing adolescent outcomes on multiple levels.

In contrast to the broader, external layers foregrounded by ecological systems theory, Bandura’s social cognitive theory focuses on the interplay among personal factors, environmental conditions, and behavior, emphasizing cognitive processes such as self-efficacy, outcome expectations, and self-regulatory mechanisms [22]. More recent applications of social cognitive theory in adolescence have highlighted how parental practices shape young people’s perceived ability to cope with challenges, regulate emotions, and engage in adaptive behaviors [23].

When viewed through the lens of social cognitive theory, parental overprotection associated adolescents’ internal processes in critical ways. Overprotective parents often dictate decisions or solve problems on behalf of their children, potentially signaling to adolescents that they are incapable of independent mastery [24]. As a result, adolescents internalize low self-efficacy beliefs and may become overly reliant on external guidance or validation [25]. Additionally, from an observational learning standpoint, repeated exposure to parental anxiety or excessive worry can lead adolescents to adopt similar patterns of perceiving and responding to stressors, further heightening the risk of depressive outcomes [26]. Hence, social cognitive theory reveals the micro-level cognitive and emotional mechanisms by which parental overprotection could undermine adolescents’ psychological well-being.

In this study, subjective well-being, conceptualized as emotional well-being, is defined following Diener as comprising individuals’ life satisfaction and positive affective experiences [27]. Subjective well-being has been widely recognized as a crucial indicator of mental health, distinct from psychological and social well-being constructs [28,29]. Therefore, this research specifically investigates subjective well-being’s role within the proposed mediation framework.

Although subjective well-being and depressive symptoms represent distinct dimensions of mental health, research has demonstrated their interconnected yet distinguishable roles [30,31]. Specifically, subjective well-being can serve as a protective factor that mitigates depressive symptoms by enhancing positive emotional resources and coping abilities [32]. Therefore, examining subjective well-being as a mediator between parental practices and depressive symptoms can help clarify protective mechanisms within the broader mental health framework.

In combining these two theoretical perspectives, the current study proposes a chain mediation model wherein parental overprotection influences adolescent depressive symptoms through two interconnected psychological processes: psychological control and adolescent well-being. The current study proposes a chain mediation model wherein parental overprotection is associated with adolescent depressive symptoms through two interconnected psychological processes: psychological control and adolescent well-being. Psychological control may restrict adolescents’ autonomy and foster dependency, both of which have been associated with an increased risk of depression [33]. Furthermore, diminished well-being may relate to decreased resilience and greater susceptibility to depressive outcomes [34]. Thus, guided by ecological systems theory, social cognitive theory, and empirical evidence, the proposed model examines how parental overprotection, psychological control, and well-being are interrelated and associated with adolescent depression.

### 1.2. Parental overprotection and student depression

Parental overprotection refers to excessive parental attention and interference that limit a child’s autonomy and independence [6]. Parker and colleagues first introduced this concept in their study of families with patients suffering from depression, suggesting that overprotective parents excessively monitor and control their children’s behavior, restricting their ability to make independent decisions and explore [35]. Research has consistently shown that parental overprotection is associated with various psychological issues in children and adolescents, including anxiety, depression, and social withdrawal [36].

Research has demonstrated a significant positive correlation between parental overprotection and adolescent depressive symptoms. Overprotective parents often display excessive concern for their children’s safety and well-being, which restricts the development of autonomy and independence. This can lead to a lack of coping skills and self-confidence when adolescents face challenges and stress [37]. Such parenting styles may foster feelings of helplessness and dependence in children, heightening the risk of emotional distress [25]. For instance, Thomasgard and Metz found that overprotective parents excessively intervene in their children’s lives, limiting independent activities. This restriction results in lower self-efficacy and higher levels of anxiety and depression when adolescents encounter stress and frustration [6]. Additionally, research suggests that overprotective parenting can impair social interactions among adolescents, further aggravating depressive symptoms [38]. Cho et al. discovered a significant association between parental overprotection and adolescent social withdrawal behaviors, which are closely linked to depressive symptoms [39]. Parker et al. also noted that overprotective parents tend to strictly regulate their children’s social activities, limiting peer interactions and increasing feelings of isolation within social support systems, which further intensifies depressive emotions [40].

### 1.3. The mediating role of psychological control and well-being

Psychological control refers to parental behaviors that manipulate, reject, or control a child’s emotions and behaviors in order to make them comply with parental expectations [41]. Parents who employ psychological control often use tactics such as shaming, inducing guilt, and emotional withdrawal to limit their child’s autonomy. Research indicates that psychological control mediates the relationship between parental overprotection and adolescent depression [42]. Specifically, parents who use psychological control may restrict their child’s independence, heightening emotional distress and psychological pressure, which in turn leads to increased depressive symptoms [43]. For instance, Soenens et al. found that psychological control significantly mediates the link between parenting and adolescent depressive symptoms [44]. Additionally, parents who practice psychological control often regulate their child’s emotional expressions, limiting opportunities for the child to express negative emotions, which impairs their ability to cope with stress and frustration [45]. This emotional regulation can lead to feelings of guilt and shame, further exacerbating depressive symptoms [46]. Grolnick and Pomerantz also found that psychologically controlling parents, by manipulating their children’s emotional expressions, increase the likelihood of depression when their children face stress [47].

Well-being encompasses the positive experiences and life satisfaction individuals feel, including emotional, psychological, and social well-being [29]. Studies suggest that parenting styles significantly influence a child’s well-being. Overprotective parents may limit their child’s autonomy and exploration, leading to a lack of achievement and satisfaction, which negatively affects their overall well-being [48]. Children with lower levels of well-being often struggle to handle stress and challenges, increasing their risk of emotional distress and depressive symptoms [49]. Previous studies indicate that parenting styles can indirectly relate to adolescent depressive symptoms through the mediation of subjective well-being. For example, Schiffrin et al. demonstrated that autonomy-supportive parenting is associated with increased subjective well-being, which is, in turn, negatively related to depressive symptoms [50]. This mediation suggests subjective well-being functions as an emotional resource that may buffer the negative effects of parenting practices on adolescents’ depressive experiences. Furthermore, adolescents with lower well-being tend to experience more negative emotions and fewer positive emotions, reducing their ability to cope with stress, thereby heightening the risk of depression [51]. As Seligman noted, individuals with high levels of well-being demonstrate stronger psychological resilience and coping skills, enabling them to manage life’s stressors more effectively and thus decreasing the likelihood of depression [52].

### 1.4. The chain mediation role of psychological control and well-being

When examining the relationship between parental overprotection and adolescent depressive symptoms, the chain mediation effect of psychological control and well-being offers a more nuanced understanding. Psychological control refers to parental behaviors that manipulate, reject, or limit a child’s autonomy in order to enforce compliance with parental expectations [41]. Research has shown that psychological control is significantly associated with adolescent depressive symptoms and serves as a mediator between parental overprotection and depression [44].

Firstly, parental overprotection is often accompanied by high levels of psychological control. Parents may use emotional manipulation, guilt, and shame to restrict their child’s autonomy [46]. This control leaves children without the necessary coping strategies or independence to face challenges and stress, resulting in increased emotional distress [10]. For example, parents who use psychological control may limit their child’s social activities and decision-making abilities, fostering feelings of helplessness and dependence, which in turn leads to heightened depressive symptoms [53].

Secondly, psychological control significantly reduces a child’s well-being, which encompasses positive experiences and life satisfaction [27]. Studies have found a strong negative correlation between psychological control and well-being, as controlling parents often restrict their child’s ability to express emotions and pursue personal interests [54]. This limitation reduces positive experiences and a sense of achievement [55]. Lower well-being leaves children less equipped to handle stress and challenges, further increasing their vulnerability to depression [56]. Moreover, well-being mediates the relationship between parenting styles and adolescent mental health. For instance, Grolnick and Pomerantz found that children with lower well-being tend to experience more negative emotions and depressive symptoms when facing stress [47]. Schiffrin et al. further demonstrated that well-being plays a key role in mediating the effects of parental control on adolescent mental health [50].

Subjective well-being is considered a mediating variable because it reflects emotional resources such as life satisfaction and positive affect, which can mitigate the impact of adverse parenting practices on adolescent depressive symptoms. Fredrickson’s broaden-and-build theory suggests positive emotional experiences broaden adolescents’ cognitive and emotional coping resources, helping buffer against stress and reducing vulnerability to depression [32]. Therefore, subjective well-being may serve as a critical intermediate variable explaining how parenting practices, including parental overprotection and psychological control, relate to adolescent depressive outcomes [50].

In summary, parental overprotection increases psychological control, which in turn reduces well-being and ultimately leads to higher levels of adolescent depressive symptoms [57]. This chain mediation underscores the complex psychological mechanisms within family dynamics and offers valuable theoretical insights for understanding and addressing adolescent depression. To reduce depressive symptoms, parents should aim to decrease overprotection and psychological control, while promoting autonomy, independence, and well-being in their children [58].

### 1.5. The current study

The current study aims to examine the complex relationship between parental overprotection and adolescent depressive symptoms, with a focus on the chain mediation roles of psychological control and well-being. Based on Bronfenbrenner’s ecological systems theory and Bandura’s social cognitive theory [16,22], the study proposes a model that explores how parental overprotection can affect adolescent depression through increased psychological control and reduced well-being. Using survey data from 823 adolescents aged 10–14, the study investigates both the direct and indirect effects of parental overprotection on depression. The findings are expected to deepen the theoretical understanding of how parenting styles associated adolescent mental health and provide practical insights for designing effective interventions to reduce depressive symptoms in adolescents. Specifically, the study highlights the importance of reducing overprotective behaviors, minimizing psychological control, and promoting adolescents’ autonomy and well-being to mitigate the risk of depression.

## 2. Methodology

The research was conducted in accordance with the principles stated in the Declaration of Helsinki for experiments involving humans. After obtaining approval from an IRB office in China (2024−27), a written parental consent form was sent to the principals of two different primary schools in Sichuan Province via email, requesting them to distribute the consent forms to the parents of potential participants. The study was conducted in three different elementary schools in Sichuan, and only the data from students whose parents had signed the informed consent form were included in the analysis. The informed consent forms were paper-based, and no rewards were provided to the participating families. The research completed between May 10, 2024, and May 20, 2024.

### 2.1. Participants

A convenience sampling method was used to survey 823 adolescents, yielding a 94% response rate. The sample consisted of 422 males (51.3%) and 401 females (48.7%), aged 10–14, with an average age of 11.95 ± 1.12 years. Informed consent was obtained from both students and their guardians prior to participation, and ethics approval was granted. All personal data were kept confidential.

Given the use of convenience sampling, we employed Harman’s single-factor test and examined demographic characteristics to preliminarily evaluate potential selection bias. Nonetheless, the implications of selection bias should be considered when interpreting the generalizability of the findings.

### 2.2. Instruments

The Center for Epidemiologic Studies Depression Scale (CES-D10) [59] developed by Andresen, was used to assess adolescents’ depression levels. The scale consists of 10 items across three dimensions: physical symptoms, depression, and positive emotions. Responses are rated on a 4-point scale, with higher scores indicating greater levels of depression. In this study, the CES-D10 demonstrated good internal consistency, with a Cronbach’s alpha coefficient of 0.809.

The study adapted the Chinese version of the Parenting Styles Questionnaire developed by Jiang et al. [60] to measure the parental overprotection. The parental overprotection dimension consisted of 8 items from the overprotection subscale, measured on a 4-point Likert scale, with higher scores indicating higher levels of parental overprotection. In this study, the Cronbach’s alpha for this scale was 0.800.

The psychological control questionnaire, based on the work of Shek et al. [61], assesses psychological control across dimensions such as emotional invalidation, excessive control, withdrawal of love, suppression of verbal expression, and personal attacks. It uses a 5-point Likert scale, with higher scores indicating higher levels of psychological control. In this study, the Cronbach’s alpha for this scale was 0.943, indicating excellent reliability.

Student Well-being Scale. The study employed the Student Well-being Scale developed by Zhao et al. [62], measuring adolescents’ subjective well-being, specifically assessing affective-emotional and cognitive-evaluative dimensions. The scale used in this study was a shortened 5-item version, selected based on Zhao et al.’s validation study from the original 14-item version. Items were rated on a 5-point Likert scale from 1 (never) to 5 (always), with higher scores indicating greater subjective well-being. The Cronbach’s alpha for this scale was 0.948, demonstrating excellent internal consistency.

### 2.3. Data processing and statistical analysis

SPSS 26.0 and the PROCESS macro [63] were used for data analysis. Harman’s single-factor test was employed to check for common method bias, while Pearson correlation was used to examine the relationships among the major variables. Additionally, the chain mediation effect of psychological control and well-being was assessed using Model 6 in the PROCESS macro, with 5,000 bootstrap resamples and a 95% confidence interval. A mediation effect was considered significant if the confidence interval did not include zero [63].

Additionally, confirmatory factor analysis (CFA) was conducted using Mplus 8.4 [64] to formally test the tau-equivalence assumption for the measurement items. Tau-equivalence refers to the condition wherein each item equally contributes to the latent construct, thereby justifying the use of simple averages [65]. Standard fit indices were used to assess the measurement model’s tau-equivalence.

## 3. Results

### 3.1. Common method bias test

Harman’s single-factor test revealed that seven factors had eigenvalues greater than 1, and the variance explained by the first factor was 36.98%, which is below the 40% threshold. This result suggests that common method bias was not a significant issue [66], allowing for the continuation of data analysis.

### 3.2. Descriptive statistics and correlation analysis of variables

Table 1 presents the descriptive statistics and correlation analysis of the variables. Parental overprotection was significantly and positively correlated with student depression. Additionally, parental overprotection showed a significant positive correlation with parental psychological control. Psychological control was positively correlated with student depression, while it was negatively correlated with well-being.

**Table 1:** Descriptive statistics and correlation analysis results [n = 566].

Variable	*M ± SD*	1	2	3	4	5
1.Gender	—	—				
2.Age	11.952 ± 1.118		—			
3. Student Depression	0.897 ± 0.607	0.135*	0.098**	—		
4. Student Well-being	4.476 ± 1.323	0.124*	−0.058	0.719**	—	
5. Psychological Control	1.842 ± 0.939	−0.029	0.046	0.484**	−0.532**	—
6. Parental Overprotection	1.901 ± 0.524	−0.049	−0.083*	0.397*	−0.393**	0.631**

Note: *p < 0.05, **p < 0.01, ***p < 0.001.

### 3.3. Direct effect test between parental overprotection and student depression

Regression analysis results showed that parental overprotection has a significant positive predictive effect on student depression (β = 0.575, t = 12.399, p < 0.001).

### 3.4. Chain mediation test of parental psychological control and well-being

**Fig 1:**
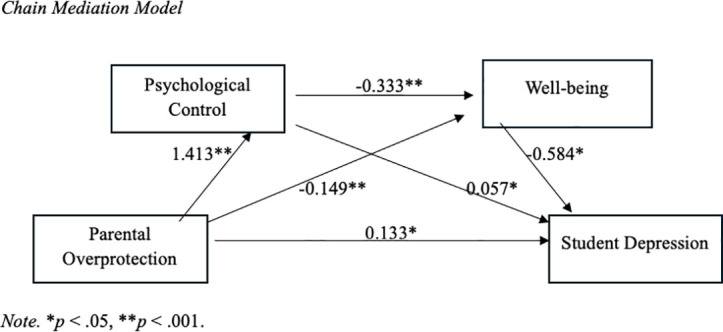
*Chain Mediation Model*. *Note.* *p < .05,**p < .001.

First, the direct effect of parental overprotection on adolescent depression was tested. The results indicated that parental overprotection significantly and positively predicts levels of adolescent depression. Following this, the chain mediation effect of parental psychological control and well-being was examined. The regression analysis (as illustrated in Fig 1) showed that parental overprotection significantly and positively predicts psychological control (β = 1.413, *t* = 23.319, *p* < 0.001) and significantly and negatively predicts well-being (β = −0.149, *t* = 2.495, *p* < 0.001). Additionally, psychological control significantly and positively predicts student depression (β = 0.057, *t* = 2.635, *p* = 0.008), while well-being significantly and negatively predicts student depression (β = −0.584, *t *= −22.591, *p* < 0.001). Moreover, psychological control significantly and negatively predicts well-being (β = −0.333, *t* = −12.422, *p* < 0.001). When both psychological control and well-being were included as mediators in the regression model, parental overprotection continued to significantly and positively predict student depression (β = 0.133, *t* = 2.977, *p* = 0.003).

Using SPSS 26.0 and Model 6 of the PROCESS macro [63], the chain mediation effect of psychological control and well-being was analyzed. The results of the mediation analysis (as shown in Table 2) revealed the following: first, the pathway from parental overprotection → psychological control → student depression, showed a mediation effect with a confidence interval that does not include 0, indicating a significant mediation effect. this pathway accounted for 13.9% of the total effect; second, parental overprotection → well-being → student depression, showed a mediation effect with a confidence interval that does not include 0, indicating a significant mediation effect. this pathway accounted for 15.1% of the total effect; third, parental overprotection → psychological control → well-being → student depression showed a mediation effect with a confidence interval that does not include 0, indicating a significant mediation effect. this pathway accounted for 47.8% of the total effect.

**Table 2:** Mediation effect analysis of psychological control and well-being in the relationship between parental overprotection and student depression.

		Estimation	Effect Size	95% Confidence Interval
Direct Effect	Parental Overprotection → Student Depression	0.132	22.9%	[0.045, 0.220]
	Parental Overprotection → Psychological Control → Student Depression	0.080	13.9%	[0.009, 0.153]
Mediation Effect	Parental Overprotection → Well-being → Student depression	0.087	15.1%	[0.009, 0.169]
	Family-school Collaboration → Parental Overprotection → Psychological Control → Well-being → Student depression	0.275	47.8%	[0.214, 0.338]
Total Mediation Effect		0.442	77.1%	[0.346, 0.545]
Total Effect		0.575		[0.484,0.666]

The results of structural equation modeling (SEM) indicated that the proposed chain mediation model demonstrated acceptable fit to the data under the constraints of tau-equivalence (χ² = 1781.68, df = 486, p < .001; RMSEA = 0.057; CFI = 0.929; SRMR = 0.069). Although the chi-square test was significant, this outcome is common when analyzing large sample sizes, where minor discrepancies become statistically detectable [65]. All other fit indices—specifically, RMSEA, CFI, and SRMR—fell within recommended acceptable ranges [67,68], indicating statistical support for the tau-equivalence assumption across measurement models for the latent variables. Therefore, the overall structural paths of the proposed model were considered justified.

Additionally, multicollinearity diagnostics indicated no problematic overlap between well-being and depression constructs. Specifically, all VIF values were below 5, and tolerance values exceeded 0.2 [69]. Confirmatory factor analysis showed that the latent correlation between well-being and depression was 0.508, which is below the commonly accepted threshold of 0.85 [65], indicating good discriminant validity between the two constructs.

## 4. Discussion

### 4.1. Parental overprotection and student depression

The findings of this study support a substantial body of research highlighting the link between parental overprotection and adolescent depression. Parental overprotection, often characterized by excessive control and a lack of autonomy granted to adolescents, has been consistently associated with a variety of negative psychological outcomes, particularly depression [36]. Overprotective parenting behaviors are often motivated by a desire to safeguard children from harm, but they inadvertently stifle essential developmental processes. Adolescents need the freedom to face challenges independently in order to build resilience, autonomy, and self-regulation skills—critical factors for emotional well-being [7]. By limiting opportunities for autonomy, overprotective parents unintentionally contribute to emotional distress and a sense of helplessness, which are well-established precursors to depression [25].

Moreover, the psychological impact of parental overprotection may differ across cultural contexts. In collectivistic societies, where family interdependence and parental authority are often emphasized, the impact of overprotection might be more pronounced. These societies typically value parental control more highly, which can result in adolescents internalizing overprotective behaviors as culturally normative, despite their detrimental effects on mental health [13]. The present study builds on these insights by showing that parental overprotection exacerbates adolescent depression through multiple psychological pathways, reinforcing the need for targeted interventions that address both the behaviors and the underlying psychological mechanisms at play.

### 4.2. The chain mediation role of psychological control and well-being

This study highlights the pivotal roles of psychological control and well-being as mediators in the relationship between parental overprotection and adolescent depression. Psychological control, which refers to the use of emotionally manipulative strategies by parents to regulate their children’s emotions and behaviors, has been identified as a significant contributor to adolescent mental health issues [41]. Research consistently shows that psychological control restricts autonomy, undermines emotional development, and fosters dependency on external validation, all of which increase the risk of depressive symptoms [44].

In addition, the role of well-being as a mediator emphasizes the critical importance of emotional resources in mitigating the impact of parental overprotection. Adolescents with higher levels of well-being, which includes emotional regulation, life satisfaction, and a sense of accomplishment, are more resilient to stressors and less prone to developing depression [52]. Parental overprotection, by decreasing well-being, restricts adolescents’ ability to cope effectively with life’s challenges, thereby exacerbating depressive tendencies [50]. These findings underscore the necessity of enhancing emotional well-being in interventions aimed at adolescents exposed to overprotective parenting.

Furthermore, the interplay between psychological control and well-being demonstrates the complexity of the mechanisms through which parental overprotection impacts adolescent mental health. As psychological control diminishes adolescents’ emotional agency and self-regulation, it also depletes their well-being, creating a feedback loop that heightens vulnerability to depression [41]. Therefore, interventions that target both psychological control and well-being are crucial for addressing the long-term effects of overprotective parenting on mental health.

### 4.3. The role of psychological control and well-being

Psychological control plays a critical intermediary role in the relationship between parental overprotection and adolescent depression. As suggested by earlier studies [41], psychological control directly impacts adolescents’ emotional regulation and self-efficacy. Parents who use emotionally manipulative behaviors—such as guilt induction or emotional withdrawal—undermine their children’s ability to regulate emotions independently, contributing to higher levels of anxiety and depression [25]. The internalization of these controlling behaviors often results in a diminished sense of self-worth and an increased reliance on others for emotional validation, further escalating depressive symptoms [70].

In light of these findings, reducing psychological control in parental practices could be an effective strategy for mitigating adolescent depression. Training parents to employ more autonomy-supportive and emotionally validating strategies may foster greater emotional resilience and self-confidence in adolescents, which in turn may reduce their susceptibility to depression [71]. These interventions should be tailored to address the emotional needs of both parents and adolescents, ensuring that both parties understand the importance of emotional autonomy and self-regulation.

Well-being, as a complementary mediator, also plays a crucial role in shaping adolescents’ vulnerability to depression. Low well-being has long been linked to a range of mental health problems, including depression [49]. Adolescents who experience reduced well-being due to overprotection are more likely to struggle with emotional distress, as they lack the resources to cope with stress effectively [48]. Parental overprotection can hinder the development of a positive self-concept and a sense of life satisfaction by limiting opportunities for exploration and self-directed growth [72]. Consequently, fostering well-being through strategies such as resilience training or promoting healthy emotional expression could serve as a buffer against the harmful effects of overprotection. School-based interventions, such as mindfulness programs, could be particularly effective in enhancing adolescents’ emotional resources, empowering them to navigate challenges independently and reducing their risk of depression [73].

### 4.4. Implications

The findings of this study contribute new theoretical insights into the effects of parental overprotection, psychological control, well-being, and student depression. By demonstrating the chain mediation of psychological control and well-being, this study expands the understanding of how parenting styles associated adolescent mental health. It enriches existing research by providing empirical evidence that applies Bronfenbrenner’s ecological systems theory and Bandura’s social cognitive theory to family dynamics and mental health.

Practically, the study provides valuable guidance for education and mental health interventions [23]. The results suggest that reducing parental overprotection and psychological control, while enhancing student well-being, can effectively mitigate the risk of depression. Parent education programs should emphasize the importance of granting children autonomy, and schools should collaborate with families to develop interventions that promote emotional well-being. These findings offer practical strategies for improving adolescent mental health, with significant implications for family education, school practices, and policy development.

### 4.5. Limitation and future study

Despite its contributions, this study has several limitations. First, the use of convenience sampling may limit the generalizability of the findings [74]. Future research should employ larger, randomized samples to improve external validity. Second, the cross-sectional design of the study prevents the establishment of causal relationships [75]. Longitudinal studies are needed to better understand the long-term effects of parental overprotection on student depression. Third, self-reported data may be subject to social desirability or memory biases. Future research should incorporate multi-source data, such as teacher evaluations or psychological assessments, to increase the objectivity of the findings. Finally, this study focused primarily on psychological control and well-being as mediators but did not explore other potential moderators, such as cultural background or socioeconomic status. Future studies should include these variables to provide a more comprehensive understanding.

## 5. Conclusion

In summary, this study provides compelling evidence that parental overprotection exerts both direct and indirect effects on adolescent depression through the dual mediators of psychological control and well-being. By integrating ecological and social cognitive perspectives, the findings highlight that overprotective parenting not only stifles adolescents’ autonomy and coping capacities but also undermines positive emotional resources critical for resilience. From a theoretical standpoint, this multi-layered relationship underscores the importance of examining parenting behaviors in a broader ecological context while also recognizing the internal cognitive-emotional processes that heighten vulnerability to depression. Practically, the study’s insights point to the need for parent-focused interventions that reduce overprotective tendencies and foster healthier styles of guidance, particularly by curbing psychologically controlling tactics and actively promoting adolescent well-being. Future research should adopt longitudinal and culturally diverse designs to strengthen causal inferences and deepen our understanding of how these processes operate across different sociocultural settings. Ultimately, these findings serve as a valuable foundation for designing integrated preventive and therapeutic programs aimed at safeguarding adolescent mental health.

## Supporting information

S1 File Data. (XLSX)
